# S-Nitrosylation of parkin as a novel regulator of p53-mediated neuronal cell death in sporadic Parkinson’s disease

**DOI:** 10.1186/1750-1326-8-29

**Published:** 2013-08-28

**Authors:** Carmen R Sunico, Tomohiro Nakamura, Edward Rockenstein, Michael Mante, Anthony Adame, Shing Fai Chan, Traci Fang Newmeyer, Eliezer Masliah, Nobuki Nakanishi, Stuart A Lipton

**Affiliations:** 1 Sanford-Burnham Medical Research Institute, Del E. Webb Center for Neuroscience, Aging, and Stem Cell Research, 10901, North Torrey Pines Road, La Jolla, CA 92037, USA; 2 Department of Neurosciences and Pathology, University of California at San Diego, 9500, Gilman Drive, La Jolla, CA 92039, USA

**Keywords:** Nitric oxide (NO), S-nitrosylation, Parkin, p53, Nitrosative stress, Parkinson’s disease

## Abstract

**Background:**

Mutations in the gene encoding parkin, a neuroprotective protein with dual functions as an E3 ubiquitin ligase and transcriptional repressor of p53, are linked to familial forms of Parkinson’s disease (PD). We hypothesized that oxidative posttranslational modification of parkin by environmental toxins may contribute to sporadic PD.

**Results:**

We first demonstrated that S-nitrosylation of parkin decreased its activity as a repressor of p53 gene expression, leading to upregulation of p53. Chromatin immunoprecipitation as well as gel-shift assays showed that parkin bound to the p53 promoter, and this binding was inhibited by S-nitrosylation of parkin. Additionally, nitrosative stress induced apoptosis in cells expressing parkin, and this death was, at least in part, dependent upon p53. In primary mesencephalic cultures, pesticide-induced apoptosis was prevented by inhibition of nitric oxide synthase (NOS). In a mouse model of pesticide-induced PD, both S-nitrosylated (SNO-)parkin and p53 protein levels were increased, while administration of a NOS inhibitor mitigated neuronal death in these mice. Moreover, the levels of SNO-parkin and p53 were simultaneously elevated in postmortem human PD brain compared to controls.

**Conclusions:**

Taken together, our data indicate that S-nitrosylation of parkin, leading to p53-mediated neuronal cell death, contributes to the pathophysiology of sporadic PD.

## Background

Characterized by motor impairment and, with time, cognitive deficits, Parkinson’s disease (PD) affects ~1% of people over the age of 65 [1]. Histopathological features include loss of dopaminergic neurons, predominantly in the substantia nigra *pars compacta*, often accompanied by intracellular inclusions called Lewy bodies [2,3]. Rather than associated with a single gene mutation, more than 95% of PD cases are sporadic in nature, in some cases epidemiologically linked to pesticide exposure [4,5]. Although there are symptomatic treatments for PD patients, currently there is no effective therapy to prevent or cure this malady.

Mutations in the parkin gene have been associated with autosomal recessive juvenile PD and rare adult cases [6-9]. Parkin encodes an E3 ubiquitin ligase [9,10], and disruption in parkin-mediated protein ubiquitination may contribute in part to neuronal cell death [11-16], although other neuroprotective actions of parkin have also been reported [17,18]. In this regard, a novel role of parkin as a transcriptional repressor of p53 was recently demonstrated [19,20].

Processes leading to progression of sporadic PD remain largely unknown. Several environmental stressors, including oxidative and nitrosative stress, have been epidemiologically linked to sporadic PD [21,22]. PD-linked environmental stressors may cause alterations in parkin solubility, promoting its aggregation and compromising its protective function [23]. Additionally, oxidative or nitrosative stress can cause posttranslational modifications on parkin (sulfonation and S-nitrosylation, respectively) to disrupt its E3 ligase activity [24-26].

In the present study, we demonstrate that S-nitrosylation of parkin also decreased its activity as a repressor of p53 gene expression, and led to an upregulation of p53 mRNA and protein. Chromatin immunoprecipitation as well as gel-shift assays showed that parkin bound to the p53 promoter, and this binding was inhibited by the S-nitrosylation of parkin. Additionally, nitrosative stress, induced by pesticides that have been reported to be epidemiologically linked to PD, induced SNO-parkin and cell death in a p53-dependent manner in both cell-based and animal models. Administration of a NOS inhibitor mitigated the increase in SNO-parkin and p53 as well as neuronal cell death. Finally, in postmortem human PD brain, the levels of SNO-parkin and p53 were increased in a correlative manner. Taken together, our data indicate that S-nitrosylation of parkin contributes to p53-mediated neuronal cell death, in part underlying the pathophysiology of sporadic PD.

## Results

### Parkin is a negative regulator of p53

**Figure 1:**
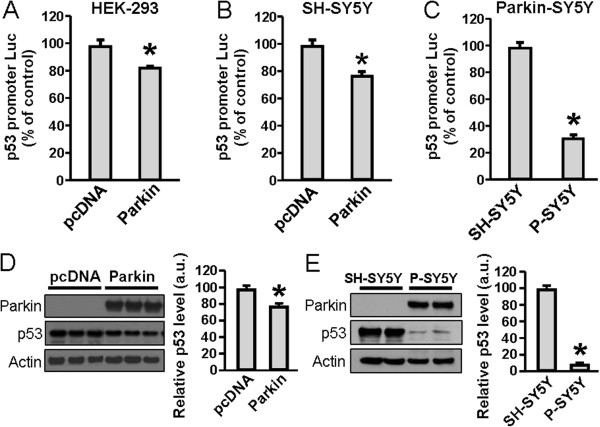
**Parkin functions as a repressor of p53 transcription and protein levels*. A***-**B**, p53 promoter activity measured by luciferase assay in HEK-293 and SH-SY5Y cells transiently transfected with parkin or the control vector pcDNA. ***C***, p53 promoter activity in SH-SY5Y cell line stably expressing parkin (P-SY5Y). ***D***-**E**, Western blot analyses of parkin, p53 and actin in SH-SY5Y cells transiently **(*D*)** or stably **(*E*)** expressing parkin. Quantification of p53 signal (right panels) was made relative to actin and expressed in arbitrary units (a.u.). All values are mean + SEM, *n* = 6 **(*A*)**, 12 **(*B*)**, 18 ***(C)***, 3 ***(D)*** and 9 ***(E)***; * *p* < 0.01.

Parkin has been reported to act as a transcriptional repressor of the p53 gene [20]. Initially, we confirmed this result by transiently expressing parkin in human embryonic kidney (HEK)-293 or human neuroblastoma SH-SY5Y cells and monitoring p53 promoter activity using a luciferase reporter assay. We found that parkin expression decreased p53 reporter gene activity in HEK-293 (Figure 1*A*) and SH-SY5Y (Figure 1*B*) cells by 16% and 22%, respectively, compared to vector-transfected control cells. Additionally, we measured p53-promoter activity in an SH-SY5Y neuroblastoma cell line stably overexpressing parkin (Parkin-SY5Y cells), and found that p53 promoter activity was decreased 68% compared to the control (Figure 1*C*). For further analysis, we quantified the effect of parkin overexpression on p53 protein levels by immunoblotting. In cells transiently overexpressing parkin, p53 protein levels were decreased by 21% (Figure 1*D*), and in cells stably expressing parkin, p53 decreased by 91% (Figure 1*E*). These experiments confirmed that exogenously expressed parkin acts as a transcriptional repressor of p53 in these experimental systems.

### S-Nitrosylation of parkin reduces its ability to repress p53 gene expression

We next asked whether S-nitrosylation of parkin affects its ability to repress p53 transcription. We initially used the neuroblastoma SH-SY5Y cells because the endogenous level of parkin expression is very low in this cell line (see Figure 1*D*), allowing us to easily identify the effect of parkin overexpression on activity. However, since parkin is not detectable in SH-SY5Y cells, this cell type cannot be used to assess the role of endogenous parkin in cell death occurring in Parkinson’s disease.

**Figure 2:**
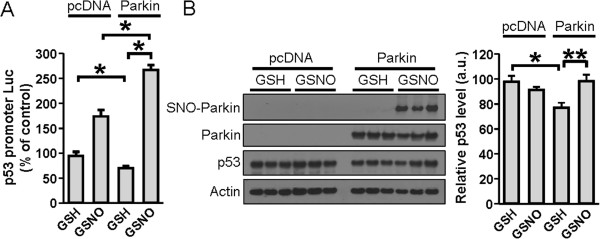
**Effect of NO on p53 transcription and protein levels in parkin-overexpressing SH-SY5Y cells. *A***, p53 promoter activity in cells transfected with the control (pcDNA) or Parkin-expression plasmid. The transfected cells were subsequently exposed to 200 μM NO donor GSNO or the control compound GSH. Values are expressed as a percentage of the control (pcDNA-transfected GSH-treated cells). ***B***, Protein levels of parkin, p53 and actin by western blot (*left panel*) in cells transiently transfected with parkin or pcDNA, and exposed to 200 μM GSNO or GSH. Quantification of p53 levels (*right panel*) was made relative to actin and expressed in arbitrary units (a.u.). Values are mean + SEM, *n* = 3; * *p* < 0.01, ** *p* < 0.05.

We transiently transfected SH-SY5Y neuroblastoma cells with control pcDNA1 (pcDNA) or parkin-expression vector, together with a vector encoding the p53-reporter. Seventy-two hours after transfection, the cells were challenged with 200 μM of the NO donor S-nitroso-glutathione (GSNO) for 6 hours. Using the biotin-switch technique, we found that parkin was S-nitrosylated under this condition (Figure 2*B*) [24,26].

Both with the pcDNA and parkin-expression vector, the cells exhibited higher levels of p53 promoter activity after GSNO exposure (Figure 2*A*), and the increase in p53 promoter activity was greater in cells expressing parkin than in control cells transfected with pcDNA. These data suggest that S-nitrosylation of parkin reduces the transcriptional repressor activity on the p53 gene. Furthermore, we performed immunoblotting and quantified the levels of p53 proteins in SH-SY5Y cells transfected with parkin-expression vector and subsequently exposed to GSNO. GSNO induced a significant increase in the p53 protein level in parkin-transfected SH-SY5Y neuroblastoma cells (Figure 2*B*). In summary, exogenous NO attenuated parkin-mediated repression of p53 transcription in SH-SY5Y cells.

### S-Nitrosylation alters nuclear localization of parkin and reduces its ability to bind to the p53 promoter

**Figure 3:**
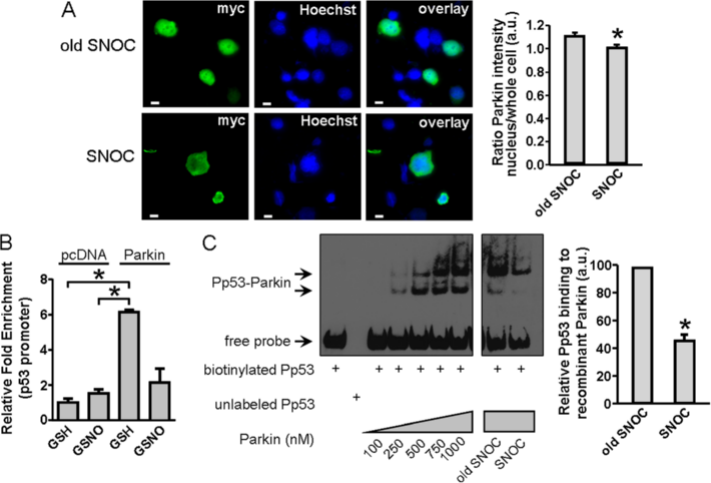
**Effect of NO on subcellular localization of parkin and its physical interaction with p53 promoter (Pp53). *A***, Histological images of SH-SY5Y cells transiently transfected with myc-tagged parkin and exposed to 200 μM old or fresh SNOC. Cells were probed with anti-myc antibody (green) and Hoechst stain (blue). Quantification of the intensity ratio of parkin in the nucleus relative to cytoplasm (*right panel*) indicates that parkin is excluded from the nucleus after SNOC exposure. Scale bar: 10 μm. Values are mean + SEM, *n* = 9 from triplicate experiments; * *p* < 0.01. ***B***, Chromatin immunoprecipitation (ChIP) assay using a specific parkin antibody (MAB5512) and oligonucleotide probe encoding the human p53 promoter sequence. Control or parkin-overexpressing SH-SY5Y cells were exposed to 200 μM GSNO (or GSH) for 4 hours prior to performing the ChIP assay. Relative fold enrichment was assessed and normalized to control. Values are mean + SEM, *n* = 4–5; * *p* < 0.05. ***C***, Electrophoretic mobility shift assay (EMSA) using recombinant parkin protein and oligonucleotide probe encoding the human p53 promoter sequence. Parkin proteins were pre-exposed to 200 μM old or fresh SNOC prior to incubation with the oligonucleotide probe. The optical density of shifted bands was quantified and expressed as percent control. Values are mean + SEM, *n* = 4; * *p* < 0.01.

For transcriptional repressor activity, parkin must be located in the nucleus in order to bind to DNA. It is thus possible that S-nitrosylation attenuates parkin function by altering nuclear localization of the protein. To test this notion, we examined the localization of parkin in cells exposed to S-nitrosocysteine (SNOC). We transiently transfected SH-SY5Y cells with myc-tagged parkin and exposed them to 200 μM SNOC (or NO-dissipated control). Six hours later the cells were fixed, immunostained with anti-myc antibody, and stained with Hoechst. We found that parkin was localized in both the nucleus and cytoplasm in control-treated cells, while parkin was preferentially localized in the cytoplasm in cells exposed to SNOC (Figure 3*A*). These data indicate that S-nitrosylation of parkin leads to its exclusion from cell nuclei.

Using chromatin immunoprecipitation (ChIP), we studied the physical interaction between parkin protein and the p53 promoter sequence in SH-SY5Y cells. In cells overexpressing parkin compared to mock-transfected cells, we observed a significant increase in the level of parkin binding to the p53 promoter (Figure 3*B*). However, when we exposed these cells to 200 μM GSNO, we could no longer detect a physical interaction between the two proteins (Figure 3*B*). These analyses suggest that S-nitrosylation of parkin attenuates its ability to bind to the p53 promoter.

We also performed electrophoretic mobility shift assays (EMSAs) to monitor the physical interaction between parkin protein and the p53 promoter sequence *in vitro*. With increasing concentrations of recombinant parkin protein, we found an increase in the amount of shifted double-stranded oligonucleotide probe (Pp53) encoding the human p53 promoter (Figure 3*C*; left panels). Pre-incubation of parkin protein with 200 μM ‘old’ SNOC for 10 minutes prior to the binding assay did not affect parkin-Pp53 interaction, while pre-incubation with 200 μM SNOC significantly reduced the amount of mobility-shifted p53 promoter probe (47% relative to old SNOC; Figure 3*C*). These data indicate that S-nitrosylation of parkin attenuates its ability to bind to the p53 promoter region.

### NO induces p53-dependent cell death in parkin-overexpressing cells

**Figure 4:**
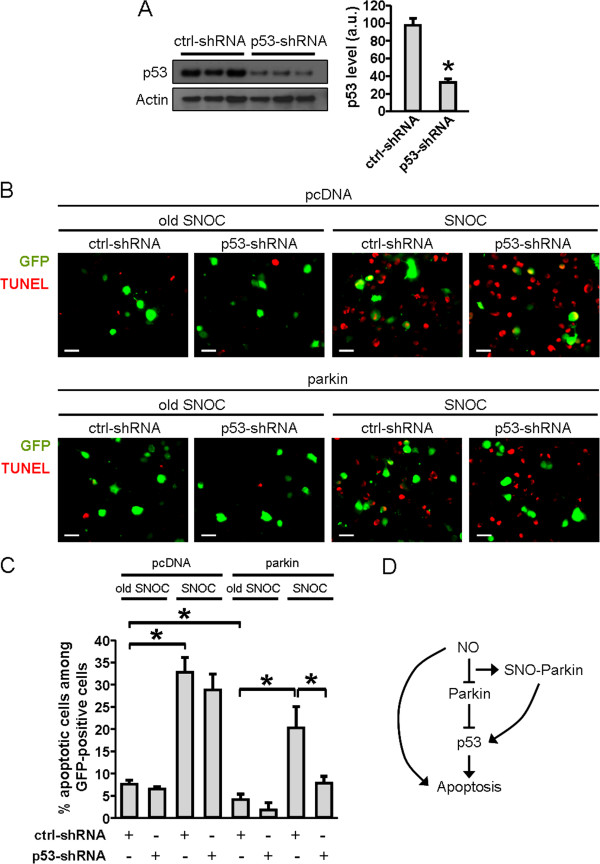
**Role of p53 in NO-induced death in SH-SY5Y cells overexpressing parkin. *A***, Validation of p53-shRNA as a gene-silencing tool. SH-SY5Y cells were transfected with control (ctrl)- or p53 shRNA encoding plasmids, and the cell lysates subjected to western blotting using anti-p53 or actin antibodies. The optical density of the p53 bands was quantified and normalized to actin, *n* = 3; * *p* < 0.01. ***B***, Histological images of SH-SY5Y cells transiently transfected with pcDNA or parkin plasmid. Cells were co-transfected with ctrl- or p53-shRNA plasmids that also encoded GFP (green). Cells were exposed to 600 μM old or fresh SNOC 24 h prior to TUNEL (red) staining. Scale bar: 20 μm. ***C***, Percentage of TUNEL-positive cells among GFP-positive cells under the designated conditions. Values are mean + SEM of 41–83 cells per condition performed in triplicate experiments; * *p* < 0.05. ***D***, Schematic representation of the pathway linking NO to apoptosis in this system.

We next asked whether S-nitrosylation of parkin contributes to p53-mediated neuronal cell death. To this end, we overexpressed parkin in SH-SY5Y cells and exposed them to a high concentration of the NO donor SNOC. In order to evaluate the involvement of p53 in this process, we performed gene silencing using a short hairpin RNA that targets human p53 (p53-shRNA). By immunoblot analysis, transient expression of p53-shRNA reduced endogenous p53 protein levels by 65% (Figure 4*A*). Considering the fact that the transfection efficiency of p53-shRNA was ~75%, we concluded that p53-shRNA effectively knocked down endogenous p53.

Next, we transiently co-transfected SH-SY5Y cells with an expression vector encoding parkin cDNA together with the p53-shRNA vector (Figure 4*B*). As controls, pcDNA and non-targeting control (ctrl-) shRNA vectors were substituted for the parkin expression vector and p53-shRNA vector, respectively. shRNA vectors also encoded GFP to facilitate identification of transfected cells. After transfection, we exposed the cells to 600 μM SNOC for 24 hours and then evaluated cell death by terminal-deoxynucleotidyl-transferase dUTP nick end labeling (TUNEL) assay. We scored the number of apoptotic cells as a percentage of total GFP-positive cells (Figure 4*C*). Firstly, transfection with the parkin plasmid reduced the number of apoptotic cells under all conditions examined, indicating that parkin overexpression was neuroprotective as expected based upon prior reports. Secondly, in cells transfected with control pcDNA, SNOC exposure induced apoptosis that was not inhibited by p53-shRNA transfection. In contrast, cells overexpressing parkin manifested SNOC-induced cell death that was significantly ameliorated by transfection with p53-shRNA. These data are consistent with the existence of a specific cell-death pathway in neuronal cells in which S-nitrosylation of parkin inactivates its neuroprotective function at least in part by triggering p53-mediated apoptosis (Figure 4*D*).

### Pesticide-induced apoptosis in parkin-overexpressing SH-SY5Y neuroblastoma cells is mediated by p53

Subsequently, we studied the mechanism of cell death induced by the herbicide paraquat (PQ) and fungicide maneb (MB), the combination of which have been epidemiologically linked to human Parkinson’s disease [27-29] and are known to induce selective loss of dopaminergic neurons in rodents [30-32]. Moreover, PQ and MB have been extensively used in both *in vitro* and *in vivo* models of Parkinson’s disease [30-34].

**Figure 5:**
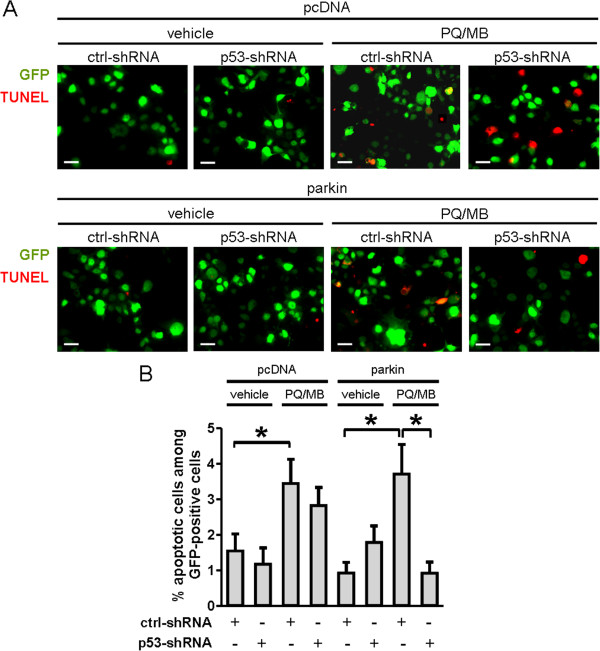
**Role of p53 in herbicide/fungicide-induced apoptosis in SH-SY5Y cells overexpressing parkin. *A***, Histological images of cells transiently co-transfected with plasmids for pcDNA or parkin, together with shRNA vectors coexpressing GFP (green). Cells were exposed to a combination of 100 μM paraquat (PQ) and 10 μM maneb (MB) or vehicle for 6 h, fixed, and subjected to TUNEL staining (red). Scale bar: 20 μm. ***B***, Percentage of TUNEL-positive cells among GFP-positive cells under the indicated conditions. Values are mean + SEM of 183–656 cells per condition performed in triplicate experiments; * *p* < 0.05.

In the present study, we transiently transfected SH-SY5Y cells with the parkin-expression vector together with the GFP-p53-shRNA vector. As described previously, pcDNA and ctrl-shRNA vectors served as controls. We then incubated the cells with 100 μM PQ and 10 μM MB for 6 hours and identified apoptotic nuclei by TUNEL assay (Figure 5*A*). We then calculated the number of apoptotic cells as a percentage of total GFP-positive cells (Figure 5*B*).

The results obtained after exposure to PQ/MB were similar to those obtained after exposure to SNOC. For example, p53-shRNA did not attenuate cell death in pcDNA-transfected cells after PQ/MB exposure. In contrast, in parkin-expressing cells, p53-shRNA abrogated PQ/MB-induced cell death, with the number of apoptotic cells returning to control values obtained in the absence of PQ/MB exposure. In summary, both SNOC and PQ/MB exposure triggered p53-dependent death in cells that were transfected with parkin.

### PQ/MB-induced neuronal cell death in primary mesencephalic cultures is mediated by NO

**Figure 6:**
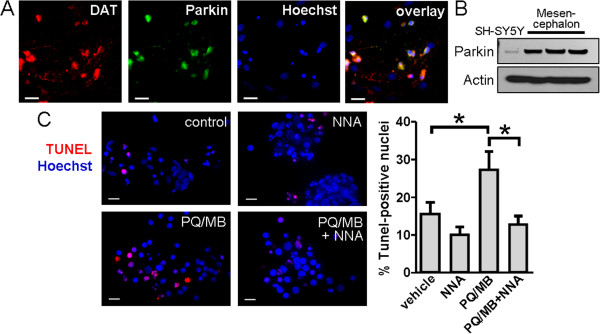
**Role of NO in herbicide/fungicide-induced neuronal death in mesencephalic primary cultures. *A***, Histological images of mesencephalic mixed cultures. Immunostaining for dopamine transporter (DAT, red), parkin (green), and Hoechst dye (blue). Scale bars: 20 μm. ***B***, Protein levels of parkin measured by western blot of cell lysates. ***C***, Images of primary cultures exposed to a combination of 100 μM paraquat (PQ) and 10 μM maneb (MB), with 1 mM N_ω_-nitro-L-arginine (NNA) or vehicle for 6 h. Staining for TUNEL (red) and Hoechst (blue). Scale bar: 10 μm. Quantification of the percentage of TUNEL-positive nuclei in each experimental condition shows PQ/MB induces cell death while NNA inhibits PQ/MB-induced death. Values are mean + SEM of 100–341 nuclei per condition performed in triplicate experiments; * *p* < 0.05.

We next studied the mechanism of PQ/MB-induced cell death in mesencephalic primary cultures, as dopaminergic neurons in this area of the brainstem are specific targets of these pesticides in PD. For this purpose, we prepared primary cultures of mesencephalon from embryonic day 13 rats. After 21 days *in vitro* (DIV), immunocytochemistry and immunoblot analyses revealed that mesencephalic cells positive for dopamine transporters (DAT) also expressed parkin (Figure 6*A,B*). We then exposed the cultured cells to 100 μM PQ and 10 μM MB for 6 hours in the presence or absence of 1 mM of the broad spectrum NO synthase (NOS) inhibitor *N*^G^-nitro-l-arginine (NNA), and found significantly increased apoptotic death with PQ/MB that was inhibited by NNA (Figure 6*C*). These results are consistent with the notion that PQ/MB-induced cell death in the mesencephalon is mediated predominantly by NO.

### SNO-parkin, p53 levels, and neuronal damage are increased in a mouse model of sporadic PD

**Figure 7:**
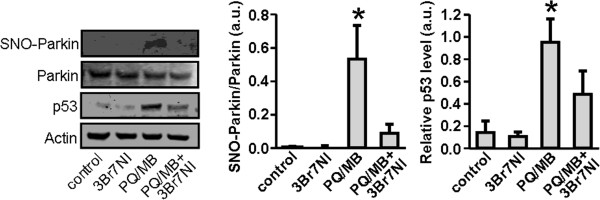
**Increased S-nitrosylation of parkin and p53 levels in a mouse model of PD.** Levels of S-nitrosylated parkin (SNO-parkin), total parkin, p53, and actin were examined by biotin-switch and western blot in mice treated with the nNOS inhibitor 3-Br-7-NI, PQ/MB, or PQ/MB with 3-Br-7-NI (*left panel*). Ratios of SNO-parkin and total parkin were quantified to indicate the extent of parkin S-nitrosylation under the indicated conditions (*center panel*). Quantifications of p53 signal were normalized to actin (*right panel*). Values are mean + SEM, *n* = 3 mice per condition; * *p* < 0.05.

We next asked whether parkin is S-nitrosylated *in vivo* in animal models of PD induced by exposure to PQ/MB in the presence or absence of the relatively neuronal specific NOS inhibitor 3-bromo-7-nitroindazole (3-Br-7-NI). Using the biotin-switch assay, we found a significant increase in S-nitrosylation of parkin (represented by the ratio of SNO-parkin/total parkin) in whole-brain lysates of PQ/MB-exposed mice compared to control brains (Figure 7). Moreover, SNO-parkin formation was attenuated by treatment with 3-Br-7-NI, indicating that endogenous NO was responsible for this nitrosylation reaction. Concomitantly, p53 expression was increased in PQ/MB-exposed animals compared to controls, and 3-Br-7-NI significantly abrogated this increase in p53 (Figure 7).

**Figure 8:**
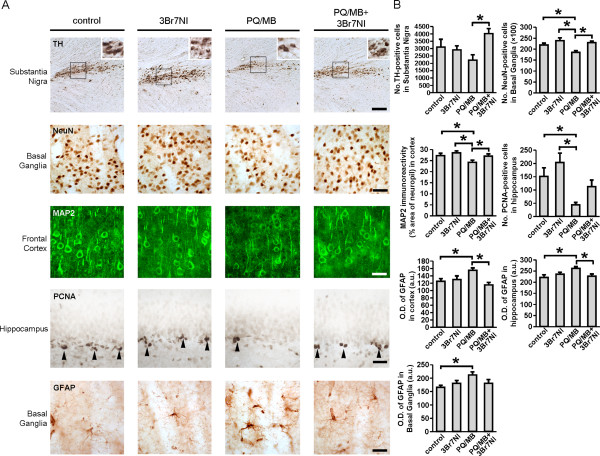
**Dopamine synthesis, neuronal survival, neurogenesis and astrocytosis in a mouse model of PD. *A***, Histological images of brain slices from mice treated with PQ/MB, the nNOS inhibitor 3-Br-7-NI, or PQ/MB with 3-Br-7-NI. Staining for TH, NeuN, MAP2, PCNA or GFAP are shown in the indicated brain regions. Scale bars: 75 μm (TH), 35 μm (NeuN), and 25 μm (MAP2, PCNA, GFAP). ***B***, Quantification of number of cells staining for TH, NeuN and PCNA; quantitative confocal immunoreactivity for MAP2; and quantitative optical density for GFAP under the conditions indicated in the specified brain regions. Values are mean + SEM, *n* = 5–6 mice per condition; * *p* < 0.05.

To determine the pathological consequences of the PQ/MB-induced nitrosative stress, we performed immunohistological analyses on tissue samples prepared from these mice. Tyrosine hydroxylase (TH) staining, representing dopaminergic neurons, was increased in the substantia nigra after 3-Br-7-NI treatment of PQ/MB-injected mice (Figure 8). Similarly, immunohistochemistry for the general neuronal markers NeuN and MAP2 revealed that PQ/MB injection caused neuronal loss in the basal ganglia and cerebral cortex, which was rescued by 3-Br-7-NI (Figure 8). Additionally, we quantified proliferating cell nuclear antigen (PCNA) staining in the dentate gyrus in order to assess progenitor cells responsible for adult neurogenesis in the hippocampus; we found that PCNA was significantly reduced after PQ/MB injection, while 3-Br-7-NI treatment partially rescued this effect (Figure 8). Reduced proliferative capacity in human PD brain has been reported previously, and these agricultural chemicals can mimic this effect in mouse PD models [35-37]. To our knowledge, however, our new findings represent the first demonstration of partial rescue of adult neural stem cell proliferation by nNOS inhibition in a PD model caused by exposure to these pesticides. Furthermore, injection of PQ/MB produced an increase in GFAP optical density in the cerebral cortex, hippocampus, and basal ganglia, consistent with previous observations that a reactive astrocytosis occurs in these rodents [30,32]; 3-Br-7-NI treatment largely inhibited this effect as well (Figure 8). Taken together, these results show that exposure to PQ/MB *in vivo* leads to S-nitrosylation of parkin, accompanied by increased p53 expression, astrocytosis, neuronal cell loss and decreased proliferation of neural stem cells, and that these adverse effects can be at least partially ameliorated by inhibition of nNOS.

### Correlation of increased SNO-parkin and p53 protein levels in human parkinsonian brains

**Figure 9:**
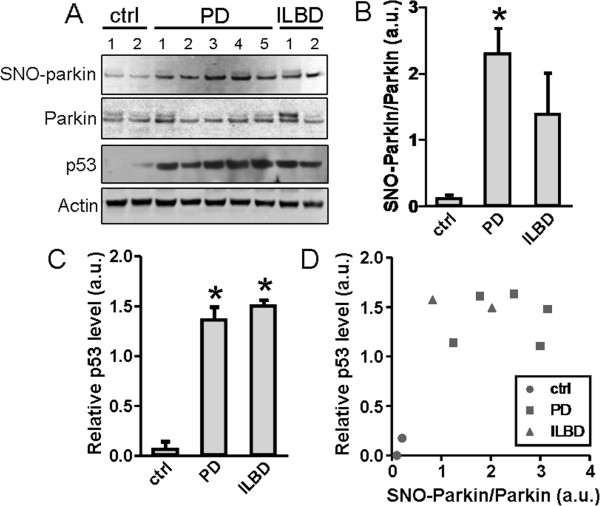
**Increased S-nitrosylation of parkin and p53 levels in postmortem human brains from Parkinson’s disease (PD) and Incidental Lewy body disease (ILBD). *A***, Levels of SNO-parkin, total parkin, p53, and actin examined by biotin-switch and western blot in lysates prepared from unaffected control brains (ctrl) and brains with PD and ILBD. ***B***, Ratios of SNO-parkin relative to total parkin were calculated to indicate the extent of parkin S-nitrosylation in ctrl, PD and ILBD samples. Values are mean + SEM of samples from 2 control, 5 PD and 2 ILBD human brains; * *p* < 0.05. ***C***, p53 levels are quantified and normalized to actin for ctrl, PD and ILBD brains. Values are mean + SEM; * *p* < 0.01. ***D***, Ratios of SNO-parkin/parkin and p53/actin were plotted for each sample. The two values are positively correlated with a Pearson correlation coefficient of 0.682 and *p* < 0.05.

Given the aforementioned results, we predicted that human PD brain would exhibit increased levels of SNO-parkin, which in turn would lead to increased p53 protein levels. To address this question, we obtained *postmortem* brains of patients with PD and incidental Lewy body disease (ILBD), along with unaffected control brains (Table 1). All brains had relatively short postmortem times and were flash frozen. We quantified the level of SNO-parkin in these brains by biotin-switch assay, and also total parkin and p53 protein levels by immunoblotting (Figure 9*A*). By quantitative densitometric analysis, we found the ratio of SNO-parkin to total parkin was increased in human PD brains by 15-fold compared to control levels (2.33 a.u. vs 0.15 a.u; Figure 9*B*). In addition, we observed a consistent increase in p53 protein levels both in the brains of PD (1.39 a.u.) and ILBD (1.53 a.u.) compared to controls (0.08 a.u.), representing an increase of >15-fold in both disease states (Figure 9*C*). Finally, the ratio of SNO-parkin to total parkin was plotted against the p53 level for each sample (Figure 9*D*), and these two values were found to be highly correlated (Pearson correlation coefficient = 0.682). Taken together, both PD and ILBD human brains exhibited an increased SNO-parkin/parkin ratio, accompanied by an increase in p53 levels.

**Table 1:** Human brain samples

**Conditions**	**Lewy body density score**	**Gender**	**Age (years)**	**PMI (hours)**
ctrl	-	m	81	2.75
ctrl	-	m	82	3.66
PD	1	m	69	4.16
PD	1	f	82	3
PD	1	m	70	1.83
PD	1	f	73	2.16
PD	3	f	85	1.75
ILBD	1	m	91	3.33
ILBD	3	m	97	1.87

Posterior temporal cortical samples from unaffected control (ctrl), Parkinson’s disease (PD) and Incidental Lewy body disease (ILBD) patients were analyzed. Each patient’s clinical condition, Lewy body density score, gender (m = male, f = female), age, and postmortem interval (PMI) are indicated.

## Discussion

Previous reports had demonstrated a novel role of parkin as a transcriptional repressor of the tumor suppressor gene p53 [20]. In agreement with this observation, in the present study we found that parkin represses p53 promoter activity in cells expressing parkin either transiently or stably. In a recent article, Duplan and colleagues identified a parkin consensus binding sequence within the promoters of p53, presenilin 1 and presenilin 2. This GCCGGAG heptamer motif [38] is encompassed in the probes used in the EMSA experiments in the current study. Moreover, we found that the effect of parkin on p53 affects cell viability. Thus, the neuroprotective action of parkin is at least in part mediated by transcriptional suppression of p53, and this action appears to be independent of parkin’s ubiquitin E3 ligase activity. Interestingly, recent studies also identified p53 as a transcriptional activator of parkin in human lung and colon cancer cell lines [39] as well as in gliomas [40]. However, in human neuronal cells, p53 does not appear to control parkin expression, and thus negative feedback regulation between parkin and p53 may not occur in neurons.

In the present work, we explore the role of a posttranslational modification, S-nitrosylation, on the neuroprotective effect of parkin that is mediated via p53 suppression. Even though exposure to NO donors can S-nitrosylate a number of other proteins, we have been able to elucidate the role of S-nitrosylated parkin by comparing results in cells with and without exogenously-expressed parkin. We report evidence that SNO-parkin attenuates the transcriptional repression of p53, and thus S-nitrosylation of parkin induces p53-mediated apoptosis in neuronal cells. Treatment with NO donors not only causes misslocalization of parkin but also inhibits the ability of parkin to bind to the p53 promoter sequence, as evidenced by EMSA and ChIP experiments. Therefore, it appears that the combination of these two effects may contribute to the cell death induced by parkin S-nitrosylation. Nonetheless, we do not rule out the possibility that the E3 ubiquitin ligase activity of parkin is also affected by nitrosative stress in PD, as indeed we and others have previously suggested [24,26]. Human sporadic PD has been epidemiologically linked to exposure to agricultural pesticides, particularly the simultaneous use of PQ and MB [4,5,27]. Our data support the notion that pesticide-induced SNO modification of parkin may contribute to ‘sporadic’ forms of PD, in a sense mimicking the effects of genetic mutations of parkin. In this regard, it should be noted that parkin-mediated control of p53 transcription is abrogated by several mutations of parkin that are known to cause familial PD [20]. In addition, S-nitrosylation, as well as tyrosine phosphorylation, of parkin have been reported to abolish its ubiquitin ligase activity in sporadic forms of PD [9,12,16,24,26,41]. Taken together, these data provide a unifying view of a mechanism underlying the pathogenesis of some forms of both sporadic and familial PD; namely, posttranslational (or epigenetic) modifications of parkin can cause partial or total loss of parkin activity, mimicking the effects of genetic mutations.

Additionally, we found that inhibition of nNOS abated the neuronal apoptosis observed in a rodent model of PD induced by exposure to PQ/MB. We also demonstrate here for the first time that inhibition of nNOS can ameliorate PQ/MB-induced reduction in adult neural stem cell proliferation. These findings are consistent with a role for NO in these events. In corroboration of this conclusion, multiple other studies have suggested the involvement of NO in the apoptotic cell death of dopaminergic and other neurons that occurs in PD [42-45]. Importantly, human postmortem brain tissue from PD patients exhibit an upregulation of NOS, reactive nitrogen species [46-48], and reactive oxygen species [10,49]. Further, in several (but not all) reports, NO inhibits adult neurogenesis in the dentate gyrus [50,51], as observed here in PD mice produced by exposure to PQ/MB. We also found that nitrosative stress induced via pesticide exposure led to S-nitrosylation of parkin in these mice, contributing to dysfunctional parkin activity. Making these observations potentially relevant to the human condition, we and others have previously reported that SNO-parkin levels are dramatically elevated in human postmortem brains from sporadic PD patients [24,26].

Furthermore, in the present study we demonstrate in neural cells expressing parkin that NO-induced death is dependent on p53. While several studies have connected nitrosative stress with p53 accumulation [52-55], S-nitrosylation of p53 does not seem to affect p53 binding to DNA [56]. In several experimental PD model systems, phosphorylation is known to activate p53 in neurons that subsequently undergo apoptosis [57-59]. Other reports have suggested a link between NO-induced processes, p53 activity and neuronal cell death, albeit the involvement of parkin was not yet known [60]. Moreover, in a number of PD model systems, inhibition of p53 has been shown to protect dopaminergic neurons and motor function [61,62], as well as increase adult neural stem cell proliferation [63]. Taken together with prior reports, our findings support the notion that p53 is a downstream effector of parkin, in part responsible for the pathogenesis of PD. Importantly, we demonstrate that S-nitrosylation of parkin leads to pathological activation of the p53 gene, thus contributing to this pathogenic mechanism.

In support of this conclusion, we found in both a murine model of pesticide-induced ‘sporadic’ PD and in human cases of sporadic PD that the brain exhibits not only elevated SNO-parkin but also an increase in p53 protein levels, which is accompanied by neuronal cell loss. While prior reports had noted increased levels of p53 in human PD brain [20,64,65], in the present study, we also found a striking positive correlation between parkin S-nitrosylation and p53 protein levels, thus supporting a possible link between these two events.

**Figure 10:**
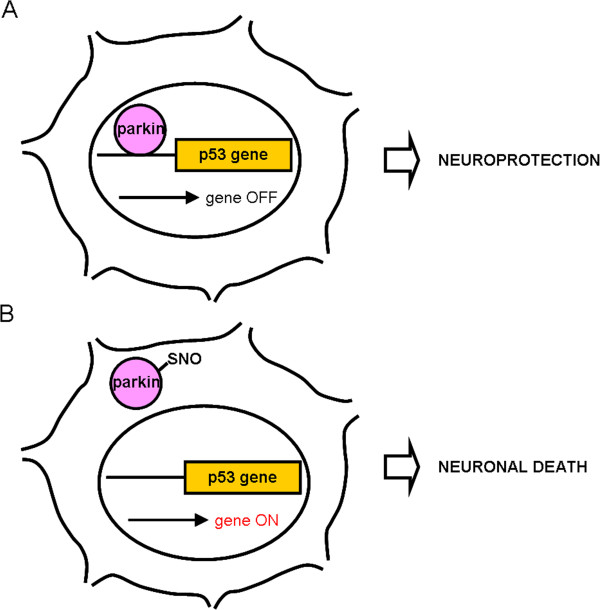
**Schematic representation of proposed mechanism whereby S-nitrosylation of parkin regulates p53-mediated neuronal death in sporadic PD. *A***, Under physiological conditions, parkin is neuroprotective by repressing p53 transcription. ***B***, During nitrosative stress, for example due to pesticide exposure, parkin becomes S-nitrosylated. SNO-parkin no longer binds to the p53 promoter and is excluded from the nucleus. This results in activation of the p53 gene and subsequent p53-mediated neuronal death.

In summary, parkin is a neuroprotective molecule that binds to the promoter region of the p53 gene, thus suppressing its activity under physiological conditions. In the present work we propose that in the presence of pesticides or other forms of environmental stress leading to the excessive generation of NO, parkin becomes S-nitrosylated. SNO-parkin, in turn, is excluded from the nucleus and loses its ability to bind to the p53 promoter sequence (Figure 10). This leads to relative activation of the p53 gene and increased levels of p53 protein, hence contributing to apoptotic cell death in affected neurons.

## Conclusions

In the present study, we show that S-nitrosylation of parkin leads to transcriptional activation of the p53 gene. This reaction may underlie, at least in part, the pathogenesis of sporadic PD. The discovery of this pathway affords an opportunity for developing novel therapeutics for PD.

## Methods

### Cell cultures, transfections and treatments

HEK-293 and SH-SY5Y cells were maintained in Dulbecco’s modified Eagle’s medium (DMEM, Sigma) supplemented with 10% of heat-inactivated fetal bovine serum (FBS, HyClone), 2 mM L-glutamine (Gibco-Invitrogen), 50 IU/ml penicillin (Omega Scientific) and 50 μg/ml streptomycin (Omega Scientific). Cells were cultured in 100 mm culture plates at 37°C in a water-saturated atmosphere of 95% air and 5% CO_2_. The SH-SY5Y cell line stably overexpressing parkin (Parkin-SY5Y cells) was prepared by selecting the transfectants with geneticin (Gibco-Invitrogen). Parkin-SY5Y cells were maintained in DMEM supplemented with 10% FBS, 2 mM l-glutamine, 50 IU/ml penicillin, 50 μg/ml streptomycin and 100 μg/ml geneticin. Transfections were performed in 6-well plates using Lipofectamine LTX and Plus Reagent (Invitrogen) according to the manufacturer’s instructions.

Cultures of neuroblastoma cell lines were grown in 6-well plates. When indicated, cells were exposed for 6–24 hours with 200–600 μM of the short-half life nitric oxide donor S-nitrosocysteine (SNOC), or control solution from which NO had been previously dissipated (designated ‘old’ SNOC). Additional exposures included 200 μM of the long-half life NO donor S-nitrosoglutathione (GSNO) or glutathione (GSH) as its negative control. The neuroblastoma cell line and mesencephalic primary cultures containing substantia nigra cells were exposed to pesticides previously linked to PD in epidemiological studies, including 100 μM of the herbicide paraquat (PQ; Fluka) and 10 μM of the fungicide maneb (MB; Fluka). These concentrations were chosen as approximately two-fold the maximal allowed exposure level from EPA data (US EPA codes CASRN 1910-42-5 and CASRN 12427-38-2, respectively). In some cases, 1 mM of the broad spectrum NOS inhibitor N^G^-nitro-l-Arginine (NNA; Alexis) was added. Cells were exposed for 6 hours at 37°C in a water-saturated atmosphere of 95% air and 5% CO_2_. Recombinant proteins were exposed to 200 μM SNOC or old SNOC for 10 min at room temperature.

### Primary mesencephalic cultures

Mixed mesencephalic primary cultures, including substantia nigra, were prepared from embryonic day-13 (E13) fetuses of Sprague–Dawley rats. The mesencephalon was dissected in ice-cold EBSS (Gibco-Invitrogen) and subsequently digested in 0.25% trypsin (Gibco-Invitrogen) for 35 minutes at 37°C in a 7% CO_2_ humidified incubator. Tissues were washed with pre-incubation medium containing DMEM Ham’s F-12 (1:1) (Omega Scientific), 10% fetal bovine serum (FBS, HyClone), 50 IU/ml penicillin (Omega Scientific) and 50 μg/ml streptomycin (Omega Scientific) and dissociated by trituration. Cells were plated on 24-well plates coated with poly-l-lysine (Sigma) in 1:1 pre-incubation medium combined with incubation medium, the latter containing DMEM Ham’s F-12, 0.5 mM l-glutamine (Gibco-Invitrogen), 1% B27 (Gibco-Invitrogen), 50 IU/ml penicillin and 50 μg/ml streptomycin, and maintained at 37°C in a 5% CO_2_ humidified incubator; 24 hours after plating, the entire medium was replaced with incubation medium. Subsequently, half of the incubation medium was replaced once every four days. At 21 days *in vitro* (DIV), cells were fixed with 4% paraformaldehyde or treated according to the specific experimental design. The percentage of dopaminergic neurons in the mixed mesencephalic primary culture was 35 ± 4.5%, as measured by immunocytochemistry for the dopamine transporter.

### Human brain tissue

Human brain tissues were provided by the Banner Sun Health Research Institute Brain and Body Donation Program. All sample groups correspond to the posterior temporal cortex, from the middle temporal gyrus at the coronal level of the end of the lateral fissure. Samples were frozen (−80°C) within less than 5 hours *postmortem* (Table 1) and homogenized with HENTS buffer, pH 7.2 (100 mM Hepes, 1 mM EDTA, 0.1 mM neocuproine, 1% Triton X-100, 0.1% sodium dodecyl sulfate) prior to biochemical analysis. The “control” group included samples from elderly patients with no history of dementia or other neurological diseases (Table 1). The “Parkinson’s disease” (PD) group included samples from 5 different brains obtained from patients whose clinical diagnosis in life was verified at *postmortem* examination (males and females, 69–85 years old; Table 1). The “incidental Lewy body disease” (ILBD) group included samples from 2 clinically normal individuals but whose autopsy revealed the presence of Lewy bodies in the central nervous system by means of α-synuclein immunohistochemistry (males, 91–97 years old; Table 1).

### Mouse model of sporadic PD from pesticide exposure

Animal care was conducted in accordance with the United States Public Health Service *Guide for the Care and Use of the Laboratory Animals*, and all experiments were approved by the University of California San Diego Institutional Animal Care and Use Committee. Exposure to the combination of pesticides paraquat (PQ) and maneb (MB) has been linked to sporadic cases of human PD in epidemiological studies [27-29] and can produce selective loss of nigrostriatal dopaminergic cell bodies and reduction in dopamine levels in the rodent striatum; the combination of paraquat and maneb has therefore been used as an experimental model of PD induced by pesticides in rodents [30-33,66]. Female mice weighing 20–30 g were injected intraperitoneally (i.p.) with freshly prepared paraquat (Fluka; 5 mg/kg) and maneb (Fluka; 15 mg/kg) twice a week for four weeks. Additional groups of animals also received the relatively specific neuronal NOS inhibitor 3-bromo-7-nitroindazole (3-Br-7-NI, Enzo Life Sciences; 30 mg/kg) or vehicle (60% DMSO in PBS) twice a week on alternate days for four weeks. Animals were sacrificed after treatment. Half of each brain was frozen at −80°C) and homogenized in HENTS buffer prior to biochemical analysis. The remaining halves were fixed in 4% paraformaldehyde for histological assessment of neuronal damage.

### Postmortem processing and immunohistochemistry

To evaluate the neurodegenerative histological alterations after pesticide exposure, blind-coded 40-μm thick vibratome sections from mouse brains fixed in 4% paraformaldehyde were immunolabeled for different markers. To assess the integrity of the dendritic system, sections were immunolabeled with the mouse monoclonal antibody against the dendritic marker microtubule-associated protein 2 (MAP2; 1:40; Chemicon, Temecula, CA), as previously described [67]. To evaluate neuronal damage, blind-coded sections were immunolabeled with mouse monoclonal antibody against the neuronal marker NeuN (1:500; Millipore, Billerica, MA). To assess astroglial response, tissue was incubated with the astroglial marker glial fibrillary acidic protein (GFAP; 1:500; Dako, Fort Collins, CO), as previously described [62,67]. For the evaluation of dopamine synthesis, sections were incubated with mouse anti-TH antibody (1:1000; Pel-Freez). To assess proliferation capacity of adult stem cells that are critical for neurogenesis, tissue was pre-treated with formamide and HCl to denature DNA. Blind-coded sections were then immunolabeled with mouse monoclonal antibody against PCNA (1:500; Santa Cruz Biotechnology, Santa Cruz, CA).

After overnight incubation with the specific primary antibody, for the detection of MAP2, sections were incubated with FITC-conjugated horse anti-mouse IgG secondary antibody (1:75, Vector Laboratories, Burlingame, CA), transferred to SuperFrost slides (Fisher Scientific, Tustin, CA) and mounted under glass coverslips with anti-fading media (Vector). The immunolabeled blind-coded sections were imaged with the laser scanning confocal microscope (MRC1024, Bio-Rad, Hercules, CA) and analyzed with the Image 1.43 program (NIH), as previously described [33,59]. For the detection of TH, NeuN, PCNA and GFAP, sections were incubated with biotinylated secondary antibody and avidin-conjugated peroxidase (both Vector). Staining was visualized by incubating in diaminobenzidine solution according to the manufacture’s directions (all from Vector Laboratories, Burlingame, CA) and analyzed with the Quantimet 570C densitometer [33].

All sections were processed under the same standardized conditions. For each mouse, a total of 3 sections were analyzed and for each section, 4 fields in the specific brain region were examined. Results for all images were averaged and expressed as the percentage area covered by immunoreactive dendrites in the case of MAP2, as number of positive neurons per square millimeter in the case of TH, NeuN and PCNA, and as corrected optical density in the case of GFAP.

### Luciferase reporter gene activity assay

Cells were seeded in 6-well plates, allowed to adhere overnight, and then co-transfected in each well with 1.5 μg of p53-responsive luciferase reporter plasmid DNA (kindly donated by Dr. Stephen H. Safe, Department of Veterinary Physiology and Pharmacology at Texas A&M University) and 0.5 μg of Renilla luciferase cDNA; in some experiments1 μg of wild-type human parkin cDNA construct was also transfected. The total amount of plasmid DNA per transfection was kept constant (3 μg per well) by balancing the total transfected DNA amount with empty vector pcDNA3 (Invitrogen). After incubation for 48 hours, cells were exposed to various protocols, according to the specific experimental design. Approximately 72 hours after transfection, cells were lysed in 1x reporter lysis buffer (Promega), 20 μl of cell extract was placed in each well of a 96-well plate, and firefly and Renilla luciferase activity were measured in a luminometer with a dual-luciferase reporter assay system following the manufacturer’s instructions (Promega). Firefly luminescence was normalized to the Renilla luminescence signal.

### Protein extraction and western blot analyses

Cultured cells were harvested and lysed in RIPA buffer (50 mM Tris–HCl pH 8.0, 150 mM NaCl, 1% Nonidet P-40, 0.5% deoxycholate, 0.1% sodium dodecyl sulphate) for 10 min at 4°C. Protein concentrations were determined using the Bio-Rad protein assay dye according to the manufacturer’s specifications. For immunoblotting, typically 5–30 μg of proteins were resolved on 4-12% polyacrylamide NuPAGE-MES gels (Invitrogen) and then wet-transferred to PVDF membranes (Millipore). Membranes were blocked in 2% skim milk in Tris-buffered saline (TBS) containing 1% Tween-20 (TBS-T). Immunoblotting was performed using mouse anti-parkin monoclonal antibody (1:1,000; MAB5512, Millipore), rabbit anti-p53 polyclonal antibody (1:1,000; 9282, Cell Signaling), mouse anti-p53 monoclonal antibody (1:1,000; SC-99, Santa Cruz), mouse monoclonal anti-β-actin antibody (1:5,000; MAB1501, Millipore), or rabbit polyclonal anti-β-actin antibody (1:5,000; 4967, Cell Signaling) for mouse samples. For cell line experiments, immunological complexes were detected with goat anti-mouse (1:3,000; Jackson) or goat anti-rabbit (1:3,000; BioRad) IgG secondary antibodies coupled to peroxidase, followed by electrochemiluminescence using the Super Signal substrate kit (Thermo Scientific) as directed by the manufacturer. For human and mouse brain lysates, goat anti-mouse at 680 LT (1:20,000; Li-Cor) or goat anti-rabbit at 800 CW (1:15,000; Li-Cor) IR-dye-conjugated secondary antibodies were used, followed by infrared analysis with Odyssey (Li-Cor). When p53 was analysed in the human neuroblastoma cell line, polyclonal antibody anti-p53 was used. In human and mouse brain samples, p53 was detected using the monoclonal antibody SC-99 (Santa Cruz).

Densitometric analysis of protein bands was performed using Adobe Photoshop. All values were normalized to their respective loading controls.

### Biotin-switch assay for detection of S-nitrosylated proteins

Analysis of S-nitrosylated parkin (SNO-parkin) with the biotin-switch assay was performed as previously described [26,68,69]. Briefly, fresh brain tissue samples were homogenized in HEN buffer, pH 7.2 (100 mM Hepes, 1 mM EDTA, 0.1 mM neocuproine) with 1% Triton X-100 and 0.1% sodium dodecyl sulfate. A total of 1 mg of protein per sample was used for the assay. Free thiol groups were blocked by incubation with 20 mM methyl methanethiol-sulfonate (MMTS; Aldrich) for 30 min at 50°C. Cell extracts were then precipitated with acetone and resuspended in HEN buffer with 1% SDS. S-Nitrosothiol groups were then selectively reduced by 75 mM ascorbate to free thiols, which were subsequently biotinylated with 4 mM N-[6-(biotinamido)hexyl]-3′-(2′-pyridyldithio)-propionamide (biotin-HPDP; Soltec Ventures). The biotinylated proteins were pulled down with streptavidin-agarose beads and analyzed by immunoblotting. Total protein as a loading control was quantified by standard immunoblot analysis. Results were expressed as SNO-parkin relative to total parkin for each sample.

### Immunocytochemistry and nuclear staining

Cell cultures were fixed in 4% paraformaldehyde in PBS for 10 min, and then permeabilized with 0.1% Triton X-100 and blocked with 5% goat serum for 30 min. Mouse anti-myc (46–0603, Invitrogen), mouse anti-parkin (MAB5512, Millipore) or rat anti-dopamine transporter (AB5990, Abcam) monoclonal antibodies were used at 1:500 dilution (4°C overnight incubation). Goat anti-mouse FITC- (115-095-166, Jackson ImmunoResearch) or goat anti-rat rhodamine red-conjugated (112-295-075, Jackson ImmunoResearch) secondary antibodies were used for fluorescence labeling at a dilution of 1:500 (RT, 1 hour). Nuclei were detected by co-staining with 12 μM Hoechst 33342 (Molecular Probes) for 30 min. Coverslips were mounted for immunofluorescence analysis using Fluoro Gel mounting medium (Electron Microscopy Sciences), and analysis of samples was performed on a deconvolution epifluorescence microscope (Axio, Carl Zeiss Microimaging GmbH).

### Terminal-deoxynucleotidyl-transferase dUTP nick end labeling (TUNEL) assay

For neuroblastoma cultures, cells were collected and TUNEL assay was performed in suspension. For primary neuronal cultures, TUNEL assay was performed on coverslips. Briefly, cells were fixed with 4% paraformaldehyde in PBS for 10 min, and then permeabilized with 0.1% Triton X-100 and 0.1% sodium citrate for 2 min. Apoptotic cells were labeled using an *in situ* cell death detection kit (Roche) according to the manufacturer’s instructions. Coverslips were mounted using Fluoro Gel mounting medium, and apoptotic cells were observed and quantified on a deconvolution epifluorescence microscope.

### RNAi-mediated knockdown of p53

psiRNA vector expressing short hairpin RNA for efficiently targeting and silencing human p53 gene (shRNA-p53) was purchased from InvivoGen (psirna42-hp53). Briefly, shRNA-p53 was produced with the psiRNA-h7SKGFPzeo plasmid from the human 7SK RNA pol III promoter, featuring a GFP-Zeo fusion gene for monitoring transfection efficiency. psiRNA plasmid targeting luciferase-GL3, whose sequence was not found in the mouse, human or rat genome databases [70,71], was used as a non-targeting control (shRNA-ctrl; InvivoGen). shRNA vectors were amplified in competent *E. coli* GT116 cells according to the manufacturer’s instructions. Transfection of neuroblastoma cells with shRNAs was performed in 6-well plates using Lipofectamine LTX and Plus Reagent (Invitrogen). A total of 3 μg of plasmid DNA per well was used for each transfection.

### Recombinant protein expression and purification

Recombinant GST-tagged parkin protein was expressed and purified as previously described [26].

### Chromatin immunoprecipitation (ChIP) assay

ChIP assays were performed using the ChIP-IT EXPRESS assay kit protocol (Active Motif, USA). Briefly, 1 × 10^7^ SH-SY5Y cells that had been transfected with pcDNA or parkin were exposed to 200 μM GSNO or GSH (as a control) for 4 hours. Cells were then cross-linked with 1% formaldehyde at room temperature for 10 min, washed with PBS and lysed, followed by centrifugation at 2400 × *g*. The nuclear pellet was resuspended in shearing buffer and sonicated with 8 pulses of 20 s each to shear DNA into 200–1000 bp fragments. Ten percent of the mixture containing protein/DNA complexes was used for “input DNA” analysis. An equal amount of the protein/DNA complex mixture was then incubated at 4°C overnight with magnetic beads and control IgG or an anti-parkin antibody (MAB5512, Chemicon International, USA). Immunoprecipitated DNA was then eluted from the magnetic beads and the cross-linking was reversed. Input and ChIP DNA were analyzed using quantitative real-time PCR. The human p53 promoter region was amplified using forward primer 5′-CTCCAAAATGATTTCCACCAA-3′ and reverse primer 5′-GGAAGCAAAGGAAATGGAGTT-3′ for determination of parkin binding. For quantitative ChIP, the PCR was performed using EXPRESS SYBR GreenER^TM^ (Invitrogen, USA) on the Stratagene Mx3000P Q-PCR system. Levels of enrichment (n-fold) were calculated using the comparative cycle threshold method.

### Electrophoretic mobility shift assay (EMSA)

A synthetic oligonucleotide covering the region of the human p53 promoter (Pp53 forward and reverse; see below) [20] labeled with biotin at the 5′ end was obtained from IDT Integrated DNA Technologies and subsequently annealed. For detection of binding of parkin to p53, 1 μM recombinant, wild-type parkin was incubated with 0.1 nM biotin-labeled DNA oligonucleotide. The specificity of the reaction was verified by incubation of Pp53 with increasing concentrations of recombinant parkin, typically from 100 nM to 1 μM. Binding reactions were performed at room temperature for 20 min in 1x binding buffer (Panomics). Then, protein-DNA complexes were resolved by electrophoresis on 6% polyacrylamide Novex DNA retardation gels (Invitrogen) at 100 V in 0.5x TBE buffer, pH 8.3 (90 mM Tris–HCl, 90 mM boric acid, 2 mM EDTA), and wet-transferred to biodyne B nylon membranes (Pall Life Sciences). Oligonucleotides were fixed to the membrane by UV crosslinking at 254 nm for 5 min. After blocking the membrane with 1x blocking buffer (Thermo Scientific), biotinylated oligonucleotides were detected by incubation with stabilized streptavidin-horseradish peroxidise conjugate (Thermo Scientific), followed by electrochemiluminescence analysis using a substrate kit (Thermo Scientific) as directed by the manufacturer.

Pp53 forward: 5′-GGCACCAGGTCGGCGAGAATCCTGACTCTGCACCCTCCTCCC CAACTCCATTTCCTTTGCTTCCTCCGGC-3′.

Pp53 reverse: 5′-GCCGGAGGAAGCAAAGGAAATGGAGTTGGGGAGGAGGGTGC AGAGTCAGGATTCTCGCCGACCTGGTGCC-3′.

### Statistical analysis

Data were quantified and stored in Excel software format (Microsoft, Redmond, WA). Graphs depicting data from quantitative analyses were generated with PRISM software (GraphPad Software). All experimental points are expressed as mean ± SEM. Statistical differences among different groups were determined by Student’s t-test (for single comparisons) or ANOVA (for multiple comparisons) using PRISM software.

## Abbreviations

GSH: Glutathione; GSNO: S-nitrosoglutathione; ILBD: Incidental Lewy body disease; MB: Maneb; NNA: N^G^-nitro-L-Arginine; NO: Nitric oxide; NOS: Nitric oxide synthase; PD: Parkinson’s disease; PQ: Paraquat; SNO: S-nitrosothiol; SNOC: S-nitrosocysteine.

## Competing interests

The authors declare that they have no competing interests.

## Authors’ contributions

CRS, TN, NN and SAL designed the study. CRS, TN, ER, MM, AA, SFC and TF-N performed relevant research and/or acquisition of data. CRS, TN, SFC, EM, NN and SAL contributed to the analysis and interpretation of data. CRS, TN, EM, NN and SAL drafted the manuscript and revised it critically. All authors read and approved the final manuscript.
